# Within-visit blood pressure variability is associated with prediabetes and diabetes

**DOI:** 10.1038/srep07964

**Published:** 2015-01-15

**Authors:** Rieko Okada, Yoshinari Yasuda, Kazuyo Tsushita, Kenji Wakai, Nobuyuki Hamajima, Seiichi Matsuo

**Affiliations:** 1 Department of Preventive Medicine, Nagoya University Graduate School of Medicine, Nagoya, Japan; 2 Department of Nephrology, Internal Medicine, Nagoya University Graduate School of Medicine, Nagoya, Japan; 3 Comprehensive Health Science Center, Aichi Health Promotion Foundation, Aichi, Japan; 4 Department of Young Leaders' Program in Health Care Administration, Nagoya University Graduate School of Medicine, Nagoya, Japan

## Abstract

We investigated the associations between within-visit blood pressure variability (BPV) and risk factors for cardiovascular disease (CVD). The study subjects included 17,795 people aged 40–74 years who underwent health check-ups in Aichi Prefecture, Japan, and completed two blood pressure measurements. Subjects were categorized into three groups according to the difference of systolic blood pressure (ΔSBP), namely, low-BPV (≤10 mmHg), moderate-BPV (11–20 mmHg), and high-BPV (>20 mmHg). Subjects were also divided into three categories as those without prediabetes (glycosylated hemoglobin A_1c_ [HbA_1c_] < 5.7%), prediabetes (HbA_1c_ 5.7–6.4%) and diabetes (HbA_1c_ ≥ 6.5% or under treatment for diabetes). The proportion of prediabetes and diabetes were significantly higher in subjects with high-BPV than in those with low-BPV after adjusting for age, sex, and mean SBP (odds ratio [95% confidence interval] was 1.16 [1.01–1.33] for prediabetes and 1.33 [1.06–1.66] for diabetes). Other CVD risk factors were not associated with high-BPV after the adjustment. In conclusion, increased within-visit BPV was significantly associated with the prevalence of prediabetes and diabetes, independent of mean SBP, in a large general population. Therefore, assessing BPV in a single visit may help to identify subjects at increased risk of impaired glycemic control.

Blood pressure variability (BPV) is now thought to be a risk factor for cardiovascular diseases (CVD)^1^,^2^. Recent data suggest that BPV persisting across several clinic visits (i.e., long-term BPV) is associated with stroke^3^,^4^, coronary heart disease, and all-cause mortality^5^. BPV measured over 24 h by ambulatory blood pressure monitoring (ABPM) (i.e., short-term BPV) is also associated with CVD events^6^,^7^. However, few studies have examined the importance of BPV measured during a single clinic visit (i.e., very-short-term BPV). Recent studies have demonstrated that within-visit BPV is associated with metabolic syndrome score^8^, target organ damage (left ventricular hypertrophy and albuminuria)^9^, and the risk of stroke^3^,^4^, but not with overall CVD or all-cause mortality^10^,^11^. If it is associated with specific CVD risk factors, within-visit BPV could be a clinically useful measure because it can be assessed in a single visit. Therefore, we sought to determine the associations between within-visit BPV and common CVD risk factors.

## Results

Overall, 312 821 subjects had complete data for CVD risk factors and had one or more BP measurement. We excluded 115 461 subjects who were taking antihypertensive drugs and/or had a history of cerebrovascular or cardiovascular diseases. We also excluded 188 487 subjects in whom only one value of blood pressure (BP) was recorded. After excluding these subjects, 17 795 subjects (6 907 men and 10 888 women) were included in the present analyses. There were no significant differences in the characteristics of subjects in whom BP was measured multiple times or once (Table 1).

The characteristics of subjects according to the BPV categories are shown in Table 2. Compared with subjects with low-BPV, those with high-BPV tended to be older, and had higher BP, glycemic levels, lipid levels, waist circumference (WC), and body mass index (BMI). The prevalence of proteinuria was also higher in subjects with high-BPV, which may be explained by the higher prevalence of diabetes. There was no difference in estimated glomerular filtration rate (eGFR) or the proportions of male/female subjects.

After adjusting for age, sex, mean systolic BP (SBP), high-density lipoprotein cholesterol (HDL-C), low-density lipoprotein cholesterol (LDL-C), triglycerides (TG), use of lipid-lowering medication, WC, BMI, and current smoking, the prevalence of prediabetes and diabetes were higher in subjects with high-BPV than those with low-BPV (odds ratio [OR] 1.16, 95% confidence interval [CI] 1.00–1.33 for prediabetes; and OR 1.39, 95% CI 1.11–1.75 for diabetes). Other CVD risk factors were not associated with high-BPV after adjusting for age, sex, and mean SBP (Table 3).

As BPV is closely related to the mean SBP, the prevalence of prediabetes/diabetes was compared in each mean SBP category. The prevalence of prediabetes/diabetes were higher in subjects with high-BPV compared to those with low-BPV in each mean SBP category (34.4% vs. 32.5%, 43.8% vs. 36.1%, and 44.0% vs. 40.7% in high-BPV vs. low-BPV for mean SBP of <120, 120–139, and ≥140 mm Hg, respectively). However, the difference was statistically significant only in subjects with mean SBP of 120–140 mm Hg after adjustment; the ORs were 1.00 (0.70–1.41), 1.34 (1.11–1.63), and 1.12 (0.92–1.37) for mean SBP of <120, 120–139, and ≥140 mm Hg, respectively, after adjustment.

## Discussion

In the present study, high within-visit BPV was significantly associated with the prevalence of prediabetes and diabetes, independent of mean SBP, in a large general population. Our results suggest that measuring BPV at a single visit may help to identify subjects at increased risk of having impaired glycemic control.

Recent data suggest that BPV is a strong prognostic factor for stroke, coronary heart disease, and all-cause mortality^1^,^2^,^3^,^4^,^5^,^6^,^7^. Several methods are available to measure BPV; visit-to-visit BPV is an index of long-term BPV, while 24-h BPV assessed by ABPM is an index of short-term BPV^1^,^2^. Both of these measurements of BPV are useful for assessing the risk of CVD^3^,^12^.

Within-visit BPV, an index of very-short-term BPV, is related to autonomic cardiovascular modulation^13^, mental stress^14^, and arterial stiffness^15^. However, few studies have evaluated the importance of BPV measured in a single clinic visit. One study revealed that within-visit BPV is associated with the risk of stroke^3^, but other studies found no association between within-visit BPV and the risks of all-cause or CVD mortality^10^,^11^. Different definitions of high-BPV might explain this discrepancy. Of note, BPV was not associated with CVD risk in studies where high-BPV was defined as the highest quintile or quartile of BPV^10^,^11^. By contrast, Rothwell et al. defined high-BPV as the highest decile of BPV^3^, and our cutoff point (20 mm Hg) is close to the 95^th^ percentile of BPV. Thus, the highest level of BPV might be associated with the risk of CVD, which should be examined in future studies.

The prevalence of prediabetes was associated with within-visit BPV in this study. Previous studies have shown that subjects with diabetes had greater 24-h BPV compared with non-diabetic subjects^16^, and glycosylated hemoglobin A_1c_ (HbA_1c_) was associated with 24-h BPV in subjects with diabetic neuropathy^17^. A recent study revealed that fasting plasma glucose was correlated with within-visit BPV^8^, but the data were not adjusted for mean SBP. To the best of our knowledge, our study was the first to show an association between prediabetes and within-visit BPV and that the association was independent of mean SBP in a large general population.

The mechanism underlying the association between prediabetes and BPV is somewhat unclear, and needs to be considered. One possible mechanism is endothelial injury caused by impaired glucose tolerance^18^,^19^. BPV is related to abnormalities in endothelial and vascular smooth muscle function, which suggests BPV may influence the pathogenesis of CVD^20^. Another mechanism could be sympathetic overactivity and autonomic imbalance initiated by impaired glucose tolerance^18^,^21^, and these changes may lead to impaired ability to control BPV^22^,^23^, as circadian BP abnormality^24^. Further studies are needed to confirm these findings and to better understand the underlying mechanisms.

Several limitations of this study should be mentioned. First, we defined prediabetes and diabetes according to HbA_1c_ rather than fasting plasma glucose. HbA_1c_ is an index of long-term glycemic control, and can be used to define prediabetes and diabetes^25^. Future studies should assess whether prediabetes defined according to fasting plasma glucose is also associated with high-BPV. Second, BPV was based on first and second measurements of BP. Although the first BP measurement is often considered unreliable^26^, a recent study suggested that the first BP measurement is related to CVD risk^8^. Additionally, it can be an easy and useful measure if difference of only two measurements of SBP reflects the risk of diabetes. Third, although mean SBP was adjusted in this study, the strong relationship between the mean SBP and BPV should be considered. The association between BPV and impaired glycemic status was observed strongly in subjects with prehypertensive range, but the association was weak in subjects with normotension or hypertension, which should be confirmed in another study. Fourth, selection bias should be considered because BP was measured twice in just 9% of the subjects who underwent health check-ups. However, there were no significant differences in the first BP measurement or other clinical factors between subjects who underwent one or multiple BP measurements. Although the guideline of this program recommends measurement of BP twice, single measurement of BP is also accepted, and it depends on each facility. Fifth, considering the cross-sectional design of this study, we could not determine the cause-effect relationship between BPV and prediabetes. Unfortunately, we lack the data on heart rate variability which reflects antonomic dysfunction, and there is no information about detailed glycemic status in the past. Whether treating subjects with prediabetes and high-BPV can prevent the progression to diabetes should be examined in longitudinal studies.

In conclusion, we have demonstrated that high within-visit BPV was significantly associated with the prevalence of prediabetes and diabetes, independent of mean SBP, in a large general population. Therefore, we consider that glycemic parameters should be monitored in subjects with high-BPV. Because BPV can be assessed in a single visit, it may be an easy and useful measure to identify subjects at increased risk of impaired glycemic control.

## Methods

### Study population

The subjects were people who underwent specific health check-ups and health guidance between April 2008 and March 2009 as part of the Tokutei-Kenshin (Special Health Check-up) programme in Aichi Prefecture, Japan. This programme was started by the Japanese government in 2008 to facilitate the early diagnosis and interventions for metabolic syndrome^27^. The target population comprises all Japanese citizens aged 40–74 years. The present study included subjects with complete data for at least two SBP and diastolic BP (DBP) measurements in one visit, and for the following CVD risk factors: HbA_1c_, HDL-C, LDL-C, TG, WC, and BMI. The guideline recommends taking a rest of ≥5 min before taking the first BP measurement and a rest of ≥1 min before taking the second BP measurement using automated machines. The first and second measurement of BP were used for the analysis because very few subjects had data on the third measurement of BP (n = 269). Serum creatinine was measured in most of the subjects. According to the program's guidelines proposed by the Japanese government, the clinician could measure either fasting plasma glucose or HbA_1c_ as an index of glycemic control. Therefore, fasting plasma glucose was unknown in most of the study subjects with complete data for the other variables listed above.

All of the subjects completed a self-administered questionnaire to document their current medications for hypertension, diabetes, and hyperlipidemia, history of cardiovascular and cerebrovascular diseases, and smoking habits (current smoker or not). BP measurement and blood and urine sampling were performed at the local medical institutions. HbA_1c_ values are presented as National Glycohemoglobin Standardization Program (NGSP) values, which were calculated with the following equation: HbA_1c_ (NGSP, %) = 1.02 × HbA_1c_ (Japan Diabetes Society, %) + 0.25%. Subjects currently taking antihypertensive drugs, and those with a history of cerebrovascular or cardiovascular diseases reported in the self-administered questionnaire were excluded from the present analyses, because antihypertensive medication influences BPV, and as the purpose of this study is to find the non-invasive methods (e.g. BPV in a single visit) to identify subjects at increased risk for cerebrovascular or cardiovascular diseases.

This study was conducted by the Aichi Chronic Kidney Disease Epidemiology Conference with support from the Aichi Kidney Foundation. Written informed consent was not required as only the existing information was used. This study was conducted in accordance with the Ethical Guidelines for Epidemiological Research by Japanese government. This study was approved by the Ethics Committee of Nagoya University School of Medicine (approval number 679).

### Definitions of BPV categories and CVD risk factors

As previous described^8^,^9^, subjects were classified into 3 groups according to the maximum minus minimum systolic BP (ΔSBP) value as low-BPV (ΔSBP ≤ 10 mmHg) moderate-BPV (ΔSBP 11–20 mmHg), and high-BPV (ΔSBP > 20 mmHg). HbA_1c_ was used as a marker of glycemic status. Subjects were divided into those without prediabetes (HbA_1c_ < 5.7%), and those with prediabetes (HbA1c 5.7–6.4%) or diabetes (HbA_1c_ ≥ 6.5% or under treatment for diabetes)^25^,^28^. Low HDL-C was defined as HDL-C < 40 mg/dL in men and <50 mg/dL in women, or the use of lipid-lowering drugs. High LDL-C was defined as LDL-C ≥ 160 mg/dL or the use of lipid-lowering drugs. Borderline-high TG was defined as TG 150–199 mg/dL and high TG was defined as TG ≥ 200 mg/dL or the use of lipid-lowering drugs^29^. Elevated WC was defined based on criteria for Asians^30^ as WC ≥ 90 cm in men and ≥80 cm in women. Using criteria for Asians^31^, overweight was defined as BMI 23.0–27.4 kg/m^2^ and obesity was defined as BMI ≥ 27.5 kg/m^2^. The eGFR was calculated using the Japanese GFR equation^32^, as follows: eGFR (mL/min/1.73 m^2^) = 194 × serum creatinine^−1.094^ (mg/dL) × age^−0.287^ (years) (×0.739 if female). Reduced eGFR was defined as eGFR < 60 mL/min/1.73 m^2^.

### Statistical analyses

We compared the characteristics of subjects between those who were included and those who were excluded from the study. We also compared the characteristics of subjects among the groups of high-BPV, moderate-BPV, and low-BPV. The prevalences of CVD risk factors in the high-BPV and moderate-BPV groups were compared with the low-BPV group. ORs and 95% CI were calculated for each CVD risk factor using unconditional logistic regression analysis adjusted for age (as a continuous variable), sex, mean SBP, HDL-C, LDL-C, TG, use of lipid-lowering medication, WC, BMI, and current smoking. HDL-C, LDL-C, TG, and use of lipid-lowering medication were not included in the analyses of HDL-C, LDL-C, and TG. WC and BMI were not included in the analyses of WC and BMI. Values of p < 0.05 in adjusted models were considered statistically significant. All analyses were carried out using STATA software version 9 (Stata Corp, College Station, TX, USA).

## Author Contributions

R.O. analyzed data and mainly drafted the article, Y.Y., K.T., K.W., N.H. and S.M. provided intellectual content of critical importance to the work described and revised the article, and all the authors approved the final version of the article to be published.

## Figures and Tables

**Table 1:** Characteristics of subjects included and excluded from the study

	Multiple BP measurement	Single BP measurement^a^
	N = 17,795	N = 188,487
Age (years)	61.7 ± 8.4	63.1 ± 8.7
Male	5239 (39.0%)	71,991 (38.2%)
Systolic BP, first-measure (mmHg)	127.5 ± 19.8	127.9 ± 17.8
Diastolic BP, first-measure (mmHg)	74.9 ± 11.8	75.3 ± 10.8
Hemoglobin A1c^b^ (%)	5.63 ± 0.57	5.60 ± 0.57
Glucose-lowering medication	648 (3.6%)	7451 (4.0%)
HDL cholesterol^c^ (mg/dl)	64.5 ± 17.5	62.4 ± 16.8
LDL cholesterol^c^ (mg/dl)	132.8 ± 32.3	131.6 ± 32.0
Triglycerides^c^ (mg/dl)	103 (74–149)	104 (75–151)
Lipid-lowering medication	1931 (10.9%)	22,534 (12.0%)
Waist circumference (cm)	82.0 ± 8.7	82.0 ± 8.9
Body mass index	22.4 ± 3.0	22.4 ± 3.1
eGFR^d^ (ml/min/1.73 m^2^)	81.1 ± 17.5	77.5 ± 15.6
Proteinurea	593 (3.3%)	8325 (4.5%)
Current smoker	2748 (15.4%)	29,836 (15.8%)

Abbreviations: BP, blood pressure; eGFR, estimated glomerular filtration rate. Data are number (%), mean ± SD, or median (inter-quartile range).

^a^These subjects were excluded due to the single measurement of blood pressure.

^b^Excludes subjects taking glucose-lowering drugs (n = 17,147 and 181,036).

^c^Excludes subjects taking lipid-lowering drugs (n = 15,861 and 165,953).

^d^Includes subjects with data for eGFR (n = 13,374 and 106,506).

**Table 2:** Characteristics of study subjects according to the level of blood pressure variability (N = 17,795)

	Blood pressure variability		
	Low-BPV	Moderate-BPV	High-BPV
	N = 13,425	N = 3272	N = 1098
Age (years)	61.4 ± 8.6	62.5 ± 7.7	63.0 ± 7.7
Male	5239 (39.0%)	1269 (38.8%)	399 (36.3%)
Systolic BP, first-measure (mmHg)	125.0 ± 18.0	133.5 ± 21.3	140.1 ± 26.9
Systolic BP, second-measure (mmHg)	124.2 ± 17.6	128.9 ± 18.8	136.7 ± 21.0
Diastolic BP, first-measure (mmHg)	73.7 ± 11.1	77.5 ± 12.4	81.7 ± 13.8
Diastolic BP, second-measure (mmHg)	73.1 ± 10.8	76.5 ± 11.6	81.3 ± 12.3
Hemoglobin A1c^a^ (%)	5.63 ± 0.57	5.64 ± 0.54	5.72 ± 0.69
Glucose-lowering medication	469 (3.5%)	120 (3.7%)	59 (5.4%)
HDL cholesterol^b^ (mg/dl)	64.6 ± 17.7	64.0 ± 16.9	64.2 ± 17.1
LDL cholesterol^b^ (mg/dl)	131.9 ± 32.2	135.0 ± 32.3	137.7 ± 33.2
Triglycerides^b^ (mg/dl)	103 (74–150)	106 (78–150)	112 (79–163)
Lipid-lowering medication	1464 (10.9%)	347 (10.6%)	120 (10.9%)
Waist circumference (cm)	81.8 ± 8.7	82.2 ± 8.6	83.3 ± 9.4
Body mass index	22.3 ± 3.0	22.4 ± 3.0	22.8 ± 3.3
eGFR^c^ (ml/min/1.73 m^2^)	81.3 ± 17.6	80.5 ± 16.9	81.3 ± 16.8
Proteinurea	422 (3.2%)	123 (3.8%)	48 (4.4%)
Current smoker	2125 (15.8%)	479 (14.6%)	144 (13.1%)

Abbreviations: BPV, blood pressure variability; BP, blood pressure; ΔSBP, maximum minus minimum systolic blood pressure; eGFR, estimated glomerular filtration rate. Data are number (%), mean ± SD, or median (inter quartile range). Low-BPV was defined as ΔSBP ≤ 10 mmHg, moderate-BPV as ΔSBP 11–20 mmHg, and high-BPV as ΔSBP > 20 mmHg.

^a^Excludes subjects taking glucose-lowering drugs (n = 17,147).

^b^Excludes subjects taking lipid-lowering drugs (n = 15,861).

^c^Includes subjects with data for eGFR (n = 13,374).

**Table 3:** Cardiovascular risk factors according to the level of blood pressure variability (N = 17,795)

	Blood pressure variability										
	Low-BPV			Moderate-BPV				High-BPV			
	N = 13,425			N = 3272				N = 1098			
	n	(%)	OR^a^ (95%CI)	n	(%)	OR^a^	(95%CI)	n	(%)	OR^a^	(95%CI)
Hemoglobin A1c											
<5.7%	8,661	(64.5)		2,072	(63.3)			630	(57.4)		
5.7–6.4%	3,830	(28.5)	1 (reference)	975	(29.8)	1.00	(0.92–1.10)	359	(32.7)	**1.16**	(1.00–1.33)
≥6.5%	934	(7.0)	1 (reference)	225	(6.9)	0.95	(0.81–1.11)	109	(9.9)	**1.39**	(1.11–1.75)
or under treatment											
HDL cholesterol											
≥40/50 mg/dl (M/F)	10,676	(79.5)		2,615	(79.9)			876	(79.9)		
<40/50 mg/dl (M/F)	2,747	(20.5)	1 (reference)	657	(20.1)	0.93	(0.84–1.03)	221	(20.2)	0.87	(0.74–1.02)
or under treatment											
LDL cholesterol											
<160 mg/dl	9,800	(73.0)		2,304	(70.4)			755	(68.8)		
≥160 mg/dl	3,624	(27.0)	1 (reference)	968	(29.6)	1.07	(0.98–1.17)	343	(31.2)	1.06	(0.92–1.22)
or under treatment											
Triglycerides											
<150 mg/dl	9,009	(67.1)		2,210	(67.5)			700	(63.8)		
150–199 mg/dl	1,493	(11.1)	1 (reference)	363	(11.1)	0.89	(0.79–1.02)	138	(12.6)	0.93	(0.76–1.14)
≥200 mg/dl	2,923	(21.8)	1 (reference)	699	(21.4)	**0.88**	(0.80–0.97)	260	(23.7)	0.93	(0.79–1.08)
or under treatment											
Waist circumference											
<90/80 cm (M/F)	8,000	(59.6)		1,892	(57.8)			563	(51.3)		
≥90/80 cm (M/F)	5,424	(40.4)	1 (reference)	1,380	(42.2)	0.94	(0.86–1.02)	535	(48.7)	1.08	(0.93–1.24)
Body mass index											
<23.0	8,274	(61.6)		1,957	(59.8)			609	(55.5)		
23.0–27.4	4,453	(33.2)	1 (reference)	1,137	(34.8)	0.96	(0.88–1.04)	401	(36.5)	0.96	(0.84–1.11)
≥27.5	698	(5.2)	1 (reference)	178	(5.4)	0.85	(0.71–1.03)	88	(8.0)	1.18	(0.90–1.53)
eGFR											
≥60 ml/min/1.73 m^2^	9,364	(92.7)		2,246	(92.4)			786	(93.9)		
<60 ml/min/1.73 m^2^	741	(7.3)	1 (reference)	186	(7.7)	1.05	(0.89–1.25)	51	(6.1)	0.86	(0.63–1.16)

Abbreviations: BPV, blood pressure variability; BP, blood pressure; ΔSBP, maximum minus minimum systolic blood pressure; eGFR, estimated glomerular filtration rate. Low-BPV was defined as ΔSBP ≤ 10 mmHg, moderate-BPV as ΔSBP 11–20 mmHg, and high-BPV as ΔSBP > 20 mmHg.

^a^Adjusted for age, sex, mean SBP, HDL cholesterol, LDL cholesterol, triglycerides, use of lipid-lowering medication, waist circumference, body mass index, and current smoking. The analyses of HDL cholesterol, LDL cholesterol, and triglycerides were not adjusted for HDL cholesterol, LDL cholesterol, triglycerides, and use of lipid-lowering medication. The analyses of waist circumference and body mass index were not adjusted for waist circumference and body mass index.

## References

[b1] Parati G., Ochoa J. E., Salvi P., Lombardi C. & Bilo G. Prognostic value of blood pressure variability and average blood pressure levels in patients with hypertension and diabetes. Diabetes Care. 36 Suppl 2S312–24 (2013). 23882065 10.2337/dcS13-2043 PMC3920798

[b2] Parati G., Ochoa J. E., Lombardi C. & Bilo G. Assessment and management of blood-pressure variability. Nat Rev Cardiol. 10, 143–155 (2013). 23399972 10.1038/nrcardio.2013.1

[b3] Rothwell P. M. *et al.* Prognostic significance of visit-to-visit variability, maximum systolic blood pressure, and episodic hypertension. Lancet. 375, 895–905 (2010). 20226988 10.1016/S0140-6736(10)60308-X

[b4] Rothwell P. M. *et al.* ASCOT-BPLA and MRC Trial Investigators. Effects of beta blockers and calcium-channel blockers on within-individual variability in blood pressure and risk of stroke. Lancet Neurol. 9, 469–80 (2010). 20227347 10.1016/S1474-4422(10)70066-1

[b5] Mancia G. *et al.* Blood pressure control and improved cardiovascular outcomes in the International Verapamil SR-Trandolapril Study. Hypertension 50, 299–305 (2007). 17606861 10.1161/HYPERTENSIONAHA.107.090290

[b6] Hansen T. W. *et al.* International Database on Ambulatory Blood Pressure in Relation to Cardiovascular Outcomes Investigators. Prognostic value of reading-to-reading blood pressure variability over 24 hours in 8938 subjects from 11 populations. Hypertension 55, 1049–1057 (2010). 20212270 10.1161/HYPERTENSIONAHA.109.140798

[b7] Kikuya M. *et al.* Prognostic significance of blood pressure and heart rate variabilities: the Ohasama study. Hypertension 36, 901–906 (2000). 11082164 10.1161/01.hyp.36.5.901

[b8] Shin J. H. *et al.* Within-visit blood pressure variability: relevant factors in the general population. J Hum Hypertens. 27, 328–334 (2013). 22971753 10.1038/jhh.2012.39

[b9] Wei F. F. *et al.* Beat-to-Beat, Reading-to-Reading, and Day-to-Day Blood Pressure Variability in Relation to Organ Damage in Untreated Chinese. Hypertension 63, 790–796 (2014). 24396027 10.1161/HYPERTENSIONAHA.113.02681

[b10] Muntner P. *et al.* Within-visit variability of blood pressure and all-cause and cardiovascular mortality among US adults. J Clin Hypertens (Greenwich) 14, 165–171 (2012). 10.1111/j.1751-7176.2011.00581.x PMC8108837 22372776

[b11] Schutte R. *et al.* Within-subject blood pressure level–not variability–predicts fatal and nonfatal outcomes in a general population. Hypertension. 60, 1138–1147 (2012). 23071126 10.1161/HYPERTENSIONAHA.112.202143 PMC3607229

[b12] Eguchi K., Hoshide S., Schwartz J. E., Shimada K. & Kario K. Visit-to-visit and ambulatory blood pressure variability as predictors of incident cardiovascular events in patients with hypertension. Am J Hypertens. 25, 962–968 (2012). 22739805 10.1038/ajh.2012.75 PMC3839090

[b13] Parati G., Saul J. P., Di Rienzo M. & Mancia G. Spectral analysis of blood pressure and heart rate variability in evaluating cardiovascular regulation. A critical appraisal. Hypertension 25, 1276–1286 (1995). 7768574 10.1161/01.hyp.25.6.1276

[b14] Webb A. J. & Rothwell P. M. Physiological correlates of beat-to-beat, ambulatory, and day-to-day home blood pressure variability after transient ischemic attack or minor stroke. Stroke 45, 533–538 (2014). 24407950 10.1161/STROKEAHA.113.003321

[b15] Liu Y. P. *et al.* Do level and variability of systolic blood pressure predict arterial properties or vice versa? J Hum Hypertens. 28, 316–322 (2013). 24152823 10.1038/jhh.2013.106

[b16] Mokhtar R. H., Ayob A. & Mohd Noor N. Blood pressure variability in patients with diabetes mellitus. Asian Cardiovasc Thorac Ann. 18, 344–348 (2010). 20719784 10.1177/0218492310375723

[b17] Liu F. *et al.* Influence of HbA1c on short-term blood pressure variability in type 2 diabetic patients with diabetic nephropathy. J Zhejiang Univ Sci B. 14, 1033–1040 (2013). 24190449 10.1631/jzus.B1300030 PMC3829652

[b18] Singleton J. R., Smith A. G., Russell J. W. & Feldman E. L. Microvascular complications of impaired glucose tolerance. Diabetes 52, 2867–2873 (2003). 14633845 10.2337/diabetes.52.12.2867

[b19] Wykretowicz A. *et al.* Endothelial function and baroreflex sensitivity according to the oral glucose tolerance test in patients with coronary artery disease and normal fasting glucose levels. Clin Sci (Lond). 109, 397–403 (2005). 15948715 10.1042/CS20050095

[b20] Diaz K. M. *et al.* Visit-to-visit and 24-h blood pressure variability: association with endothelial and smooth muscle function in African Americans. J Hum Hypertens. 27, 671–677 (2013). 23615389 10.1038/jhh.2013.33 PMC3745784

[b21] De Angelis K., Senador D. D., Mostarda C., Irigoyen M. C. & Morris M. Sympathetic overactivity precedes metabolic dysfunction in a fructose model of glucose intolerance in mice. Am J Physiol Regul Integr Comp Physiol. 302, R950–957 (2012). 22319048 10.1152/ajpregu.00450.2011 PMC3330769

[b22] Formes K. J., Wray D. W., O-Yurvati A. H., Weiss M. S. & Shi X. Sympathetic cardiac influence and arterial blood pressure instability. Auton Neurosci. 118, 116–124 (2005). 15795185 10.1016/j.autneu.2005.01.002

[b23] Su H., Wang J., Zhu Y., Wang G. & Cheng X. Discrepancy among three blood pressure readings within one measurement and relevant influencing factors. Blood Press Monit. 15, 152–157 (2010). 20186047 10.1097/MBP.0b013e328337cea6

[b24] Gupta A. K., Greenway F. L., Cornelissen G., Pan W. & Halberg F. Prediabetes is associated with abnormal circadian blood pressure variability. J Hum Hypertens. 22, 627–33 (2008). 18480832 10.1038/jhh.2008.32 PMC2573901

[b25] American Diabetes Association. Diagnosis and classification of diabetes mellitus. Diabetes Care. 33, S62–69 (2010). 20042775 10.2337/dc10-S062 PMC2797383

[b26] Bovet P. *et al.* Assessing the prevalence of hypertension in populations: are we doing it right? J Hypertens. 21, 509–517 (2003). 12640244 10.1097/00004872-200303000-00016

[b27] Mizushima S. & Tsushita K. [New Strategy on Prevention and Control of Noncommunicable Lifestyle-Related Diseases Focusing on Metabolic Syndrome in Japan.]. Asian Perspectives and Evidence on Health Promotion and Education. [Takashi Muto] [31–39] (Springer, Berlin, 2011).

[b28] Droumaguet C. *et al.* DESIR Study Group. Use of HbA1c in predicting progression to diabetes in French men and women: data from an Epidemiological Study on the Insulin Resistance Syndrome (DESIR). Diabetes Care 29, 1619–1625 (2006). 16801588 10.2337/dc05-2525

[b29] Expert Panel on Detection,. Evaluation,. and Treatment of High Blood Cholesterol in Adults. Executive Summary of The Third Report of The National Cholesterol Education Program (NCEP) Expert Panel on Detection, Evaluation, And Treatment of High Blood Cholesterol In Adults (Adult Treatment Panel III). JAMA. 285, 2486–2497 (2001). 11368702 10.1001/jama.285.19.2486

[b30] Alberti K. G. *et al.* International Diabetes Federation Task Force on Epidemiology and Prevention; Hational Heart, Lung, and Blood Institute; American Heart Association; World Heart Federation; International Atherosclerosis Society; International Association for the Study of Obesity. Harmonizing the metabolic syndrome: a joint interim statement of the International Diabetes Federation Task Force on Epidemiology and Prevention; National Heart, Lung, and Blood Institute; American Heart Association; World Heart Federation; International Atherosclerosis Society; and International Association for the Study of Obesity. Circulation 120, 1640–1645 (2009). 19805654 10.1161/CIRCULATIONAHA.109.192644

[b31] WHO Expert Consultation. Appropriate body-mass index for Asian populations and its implications for policy and intervention strategies. Lancet. 363, 157–163 (2004). 14726171 10.1016/S0140-6736(03)15268-3

[b32] Matsuo S. *et al.* Collaborators developing the Japanese equation for estimated GFR. Revised equations for estimated GFR from serum creatinine in Japan. Am J Kidney Dis. 53, 982–992 (2009). 19339088 10.1053/j.ajkd.2008.12.034

